# EEG-based finger movement classification with intrinsic time-scale decomposition

**DOI:** 10.3389/fnhum.2024.1362135

**Published:** 2024-03-05

**Authors:** Murside Degirmenci, Yilmaz Kemal Yuce, Matjaž Perc, Yalcin Isler

**Affiliations:** ^1^Department of Biomedical Technologies, Izmir Katip Celebi University, Izmir, Türkiye; ^2^Department of Computer Engineering, Alanya Alaaddin Keykubat University, Alanya, Antalya, Türkiye; ^3^Faculty of Natural Sciences and Mathematics, University of Maribor, Maribor, Slovenia; ^4^Department of Medical Research, China Medical University Hospital, China Medical University, Taichung, Taiwan; ^5^Complexity Science Hub Vienna, Vienna, Austria; ^6^Department of Physics, Kyung Hee University, Seoul, Republic of Korea; ^7^Department of Biomedical Engineering, Izmir Katip Celebi University, Izmir, Türkiye

**Keywords:** brain-computer interfaces (BCIs), electroencephalogram (EEG), feature reduction, machine learning, finger movements (FM) classification, intrinsic time-scale decomposition (ITD)

## Abstract

**Introduction:**

Brain-computer interfaces (BCIs) are systems that acquire the brain's electrical activity and provide control of external devices. Since electroencephalography (EEG) is the simplest non-invasive method to capture the brain's electrical activity, EEG-based BCIs are very popular designs. Aside from classifying the extremity movements, recent BCI studies have focused on the accurate coding of the finger movements on the same hand through their classification by employing machine learning techniques. State-of-the-art studies were interested in coding five finger movements by neglecting the brain's idle case (i.e., the state that brain is not performing any mental tasks). This may easily cause more false positives and degrade the classification performances dramatically, thus, the performance of BCIs. This study aims to propose a more realistic system to decode the movements of five fingers and the no mental task (NoMT) case from EEG signals.

**Methods:**

In this study, a novel praxis for feature extraction is utilized. Using Proper Rotational Components (PRCs) computed through Intrinsic Time Scale Decomposition (ITD), which has been successfully applied in different biomedical signals recently, features for classification are extracted. Subsequently, these features were applied to the inputs of well-known classifiers and their different implementations to discriminate between these six classes. The highest classifier performances obtained in both subject-independent and subject-dependent cases were reported. In addition, the ANOVA-based feature selection was examined to determine whether statistically significant features have an impact on the classifier performances or not.

**Results:**

As a result, the Ensemble Learning classifier achieved the highest accuracy of 55.0% among the tested classifiers, and ANOVA-based feature selection increases the performance of classifiers on five-finger movement determination in EEG-based BCI systems.

**Discussion:**

When compared with similar studies, proposed praxis achieved a modest yet significant improvement in classification performance although the number of classes was incremented by one (i.e., NoMT).

## 1 Introduction

Neuroimaging covers various direct and indirect techniques used to visualize both the structure and the function of the nervous system. These methods include MR (Magnetic Resonance Imaging), CT (Computed Tomography), PET (Positron Emission Tomography), and EEG (electroencephalography). Among them, aside from being non-invasive, EEG retains some advantages over others such as high temporal resolution, easy accessibility, and low cost. Since EEG can capture brain activity in real-time in millisecond precision, it has become popular in neuroscience research, clinical diagnostics, and BCI (Brain-Computer Interface) systems. BCIs translate neural signals into commands for controlling external devices through software applications. In recent developments, researchers have delved into analyzing EEG patterns linked to particular finger movements. BCIs engineered to decipher these patterns offer the prospect of individuals operating external devices or interfaces solely through brain activity, eliminating the necessity for physical muscle movements. This advancement holds immense promise in crafting prosthetic hands capable of individual finger control, managing numerous devices, facilitating neurorehabilitation, and extending into applications within gaming and entertainment industries (Aricò et al., 2018). In the following subsections, after a literature review on both brain-computer interfaces and state-of-the-art finger movement classification studies, we mentioned our aim, our contributions to the literature, and the structural organization of this article, respectively.

### 1.1 Brain-computer interfaces

BCIs are computer-assisted systems that record the brain's electrical signals based on different brain monitoring techniques, analyze the signals on the interface, and convert them to specific commands to control external devices such as computers, wheelchairs, and prostheses without any physical movement (Belkacem et al., 2020). Consequently, BCI technology can help people suffering from various motor disabilities such as stroke patients to communicate with the outside, and indirectly perform motor function (Wolpaw et al., 2002). Among several different neuroimaging modalities, Electroencephalography (EEG) is widely used to capture brain activities. It is preferred for designing BCI systems due to the fact that EEG has many advantages such as its high temporal resolution, non-invasiveness, easy operation, relatively low cost, and portability (Vidal, 1977; Chen et al., 2015).

EEG-based BCI systems that manipulated motor imagery signals generated through the movements of large body parts such as hands, feet, and tongue have been proposed to control assistive devices throughout the past several decades (Pfurtscheller and Neuper, 2001; Alazrai et al., 2019; Degirmenci et al., 2023). However, such systems propose only limited control dimensions for prosthetic devices, thereby, the potential of utilizing these systems to control further complex assistive devices is restricted (Sciaraffa et al., 2022). In the last decade, numerous research studies have examined the decoding of movements of fine body parts to improve such systems (Alazrai et al., 2019).

The decoding of the movements performed by various fingers of a hand may increase the control dimensions of the EEG-based BCI systems. This, in turn, might provide subjects who utilize assistive devices to better carry out numerous skillful tasks. However, the decoding of finger movements (FM) within the same hand is considered as a demanding research area among motor imagery signal analysis studies (Alazrai et al., 2019). Employing and analyzing different kinds of feature extraction methods, feature selection methods, and classification algorithms play an important role in order to improve the efficiency of EEG-based BCI systems, which analyze FM and generate relevant commands from the recorded EEG data. In the literature, various feature extraction methods, feature reduction methods, and classification algorithms have been suggested for decoding FM. Different time-domain, frequency-domain, and spatial-domain EEG features have been calculated to predict FM in the past decade. The raw EEG time series (Kaya et al., 2018; Mwata-Velu et al., 2022; Zahra et al., 2022), different amplitude-based, and statistical-based EEG signal features (Degirmenci et al., 2024) were utilized to examine the effectiveness of the time domain. As for the spectral-domain features, different frequency-domain [Fourier transform (Kaya et al., 2018)] and time-frequency domain [Wavelet transform (Yahya et al., 2019), Short-time Fourier transform (Azizah et al., 2022), Empirical mode decomposition (Mwata-Velu et al., 2021)] representation algorithms and their various versions were investigated to classify FM. Common spatial pattern (Anam et al., 2019, 2020) and its different versions (Kato et al., 2020) are one of the most experimented methods for the analysis of spatial domain in FM classification. These different extracted features have been successfully classified using various machine learning algorithms. However, it is a challenging scientific task to determine and choose the most efficient combination of these methods. Providing optimal and relevant features is important for improving classifier performance (Narin et al., 2014; Degirmenci et al., 2023). Therefore, the implementation of effective feature extraction methods and feature reduction methods is essential for facilitating the following task of machine learning algorithms.

### 1.2 State of the art for finger movement classification

In the last decade, various signal processing and classification methods have been successful in FM classification **(up to 91.70%)** applied in the classification of EEG signals for FM tasks.

Kaya et al. (2018) conducted a Support Vector Machine (SVM) based classification study to classify the five FM of a hand. In their study, they used the data set they collected from a total of eight subjects who agreed to participate. They exploited the power of EEG subbands, Fourier Transform (FT) amplitudes, and EEG time series to represent 19-channel EEG signals as features. An average accuracy of 43.00% was obtained. Moreover, a subject-dependent classification study was also carried out and the performances of eight subjects were found to vary in the range of (20.00, 60.00%).

In Anam et al. (2019), the classification of five FM for the subject-dependent condition using the EEG signals of four subjects was aimed. To this purpose, the Common Spatial Pattern (CSP) based feature extraction process was performed and the Random Forest (RF) algorithm was executed. The classification performance was found to be 100% for training accuracy for each subject and the test accuracy performances ranged between 51.00 and 56.00%.

In 2022, Azizah et al. (2022) carried out a subject-dependent FM classification study. They performed channel selection based on One vs. Rest Common Spatial Pattern (CSP-OVR) and four out of 19 EEG channels were defined as relevant channels in their study. They extracted the spectrogram features from these selected channels. Their subject-dependent experimental results showed that the accuracy in classifications employing SVM ranged from 21.20 to 66.60%.

In the study conducted by Kato et al. (2020) in 2020, a multi-class CSP and Complex Fourier amplitudes-based feature extraction process was presented. They extracted features using 19 EEG channels for FM classification. According to their subject-dependent results, the training results of classifications carried out with the SVM algorithm were reported in the range of 23.90–58.30%.

Recently, deep learning approaches from machine learning methods have been the focus of attention by researchers in many different research areas such as disease detection from medical images (Narin and Isler, 2021), emotion recognition from biological signals (Ozdemir et al., 2021) and Electrocardiography (ECG) based arrhythmia detection (Degirmenci et al., 2022a) due to the fact that these architectures provide improved performance of classification. In addition, the main reason for this is that feature extraction and classification stages can be performed together in the hidden layers of deep learning structures. Considering these structures' benefits and advantages, deep learning approaches are also included for the classification of FM and motor imagery tasks in the literature.

In 2021, Mwata-Velu et al. (2021) performed a feature extraction process based on Empirical. Mode Decomposition (EMD) using four effective EEG channels which were selected from 19 EEG channels. They performed deep learning (BiLSTM) based subject-dependent classifications for the prediction of FM. Using EMD-based feature extraction and deep learning structure, training accuracy values in eight subjects were calculated in the range of 73.47- -98.69%, and test performances were calculated in the range of 66.00–76.13%.

In another study conducted in 2022, Mwata-Velu et al. (2022) worked on the classification of EEG time series with deep learning (EEGNet) structure. EEG signals of four subjects were used from a dataset that included EEG data of eight subjects, and at the same time, four out of 19 EEG channels were selected for their suggested study. In the subject-dependent analyses performed with four subjects, training successes were reported in the range of 80.10–91.70%.

In Anam et al. (2020), an FM classification study, a model that uses CSP algorithm-based feature extraction and Autonomous Deep Learning (ADL) based classification was proposed. They used 19-channel EEG signals from four subjects for their experimental process. With respect to the subject-dependent classifications, training performances ranged from 74.73 to 77.61%, and test performances ranged from 74.61 to 77.75%.

In another related paper Zahra et al. (2022), which was published recently in 2022, the performance of a Convolutional Neural Networks (CNN) was evaluated based on an original study design. In their model, EEG time series were combined with sliding window (Dietterich, 2002) and noise enhancement (Mitaim and Kosko, 1998) methods to extract the features. They obtained the features from 19-channel EEG signals of eight subjects. They conducted a subject-independent FM classification and achieved a training accuracy of 57.50%.

In their study conducted in 2022, Limbaga et al. (2022) carried out a CNN (EEGNet) based study for feature extraction and signal classification of five motor imagery classes of a hand. They reinforced their suggested model using a transfer learning approach through an EEG data set that includes 19-channel EEG signals of eight subjects. They reduced the EEG channel number to 14 and utilized the EEG signals of only four subjects. In addition to this data set, they recorded EEG signals from a subject while the subject imagined five different hand positions. According to their subject-independent evaluations, they achieved an accuracy of 51.74% success with the transfer learning model which is a reinforced model.

When the studies mentioned above that aimed to classify the motor imagery tasks of FM of a hand are examined, it was observed that the performances remained at relatively low rates in studies using all EEG channels and in subject-independent classification studies. The studies showed that the performances got higher with channel selection-based and subject-dependent classifications. The cause for the low level of performance in the classification of FM may be that the movements of the fingers on a hand are actually controlled from the same region of the motor cortex (Kaya et al., 2018). Kaya et al. (2018) investigated the event-related potential (ERP) curves of motor imagery tasks of other body limb movements together with motor imagery tasks of FM. They reported that the curves could not be clearly differentiated in the motor imagery tasks of FM. Therefore, there is a need to increase the classification performance by using effective feature extraction methods, feature selection methods, and classification algorithms for the classification of FM tasks.

### 1.3 The aim of the study

In 2007, an iterative signal decomposition technique, which is known as intrinsic time-scale decomposition, (ITD) was introduced to analyze nonlinear or non-stationary signals (Frei and Osorio, 2007). Recent studies have performed ITD-based approaches for the analysis of biomedical signals. ITD-based feature extraction processes were conducted in various EEG-based studies for different objectives such as epilepsy detection (Martis et al., 2013; Degirmenci and Akan, 2020), and attention deficit hyperactivity disorder (ADHD) recognition (Karabiber Cura et al., 2023). Considering its ability to discriminate different classes, we studied to explore whether ITD promises superior use, or not, in classifying other biomedical signals.

In this study, therefore, we suggest new praxis for the classification of FM tasks using ITD of EEG signals. The different modes that are defined as Proper Rotation Components (PRCs) and their combinations are acquired through ITD. Various features are evaluated using only modes and their combinations. In addition to the ITD-based feature extraction process, the effectiveness of statistically significance-based feature selection (ANOVA) is also investigated. The extracted ITD-based features are classified by eight different machine learning algorithms (Decision Tree, Discriminant Analysis, Naive Bayes, *K*-Nearest Neighbors, Support Vector Machine, Ensemble Learning, Neural Networks, and Kernel Approximation). Different performance evaluation metrics are employed for the accurate evaluation of the outputs of the suggested study.

### 1.4 Contributions

The novel contributions of this research study are summarized as follows:

- The classification of EEG signals of FM tasks is presented, using the **ITD signal decomposition, and various feature extraction methods**.
- Modes extracted by the ITD are utilized to evaluate several features, including **Power, Mean, Sample Entropy, High-Frequency Moments (First Moment, Second Moment, Third Moment, Fourth Moment), and Hjorth Parameters (Activity, Mobility, Complexity)**.
- The first **3 modes ({PRC1},{PRC2}, and {PRC3}), and different combinations of them ({PRC1,PRC2},{PRC1,PRC3},{PRC2,PRC3}, and {PRC1,PRC2,PRC3})** are used for feature extraction and the effectiveness of only modes and their combinations are investigated with different machine learning algorithms, separately.
- The investigation of an appropriate and sustainable **machine learning model** for the proposed features to differentiate the FM tasks, and improve classification performance (success rate) as compared with the existing methods.

Finally, it must also be noted that this is the **first study** with a model that brings different combinations of PRCs extracted by ITD and various other features to classify FM tasks, to the best of our knowledge.

### 1.5 Paper organization

The rest of the paper is organized as follows: The EEG dataset used in this study, and EEG signal analysis methods are performed by the proposed ITD method which are ITD-based feature acquisition, statistical significance-based feature selection, classifier algorithms, and performance evaluation metrics are presented in Section 2. Experimental results are given in Section 3 and the results of the proposed approaches are discussed in Section 4. The outcomes of the study are summarized in Section 5.

## 2 Materials and methods

**Figure 1:**
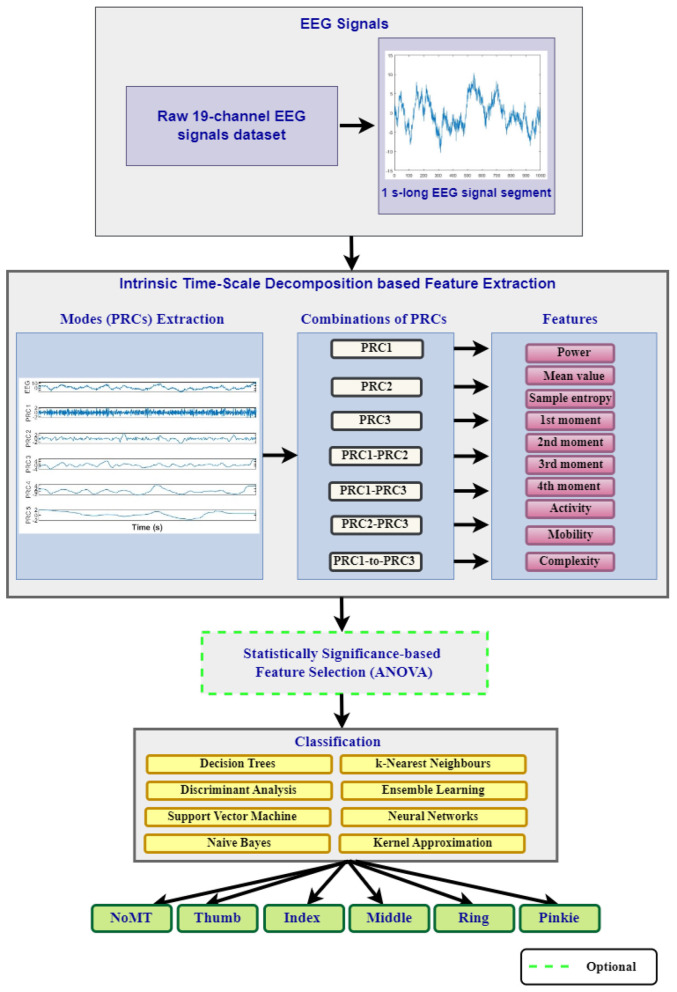
The block diagram of the study.

This study design mainly consists of five stages that are described in Isler (2009). These are EEG Data Acquisition, ITD-based Feature Extraction, Feature Reduction, Classification, and Performance Evaluation. The processes performed in each stage were delineated with details in the sub-headings. Out of these five stages/steps, the first four stages constitute the proposed classification model. Figure 1 shows the block diagram for the proposed model with its stages/steps.

### 2.1 EEG dataset description

In this study, the EEG dataset, which is a large electroencephalographic motor imagery dataset for EEG-based BCIs, presented by Kaya et al. (2018) is benefited. The dataset consists of motor imagery EEG signals that were recorded from 13 healthy subjects through 19 channels. 19 EEG electrodes together with two reference electrodes and the ground electrode were placed according to the international 10/20 EEG electrode placement system. The researchers reported that they recorded the EEG signals using an EEG-1200 JE-912A system. They performed an individual motor imagery experiment based on the movements of 10 different body limbs for four different BCI interaction paradigms. Among these planned paradigms, Paradigm #1-(CLA), which means classical left/right-hand motor imagery includes three imageries, and these are left and right-hand movements, and one passive mental imagery in which subjects remained neutral in no motor imagery. Paradigm #2-(HaLT), which means hand/leg/tongue motor imagery contains six tasks, and it is an extended version of the 3-state CLA paradigm with motor imagery tasks of right and left foot movement and tongue movement. Paradigm #3 (5F), which means 5-finger motor imagery includes FM imageries of the five-finger movement of a hand. During the tasks given for different fingers, subjects implemented the corresponding imageries invoking as flexion of the relevant finger up or down. Finger movement imageries were coded as follows: Thumb (Class 1), Index finger (Class 2), Middle finger (Class 3), Ring finger (Class 4), and Pinkie finger (Class 5). Paradigm #4 (NoMT), which means no imagery, visual stimuli only is the case in which no visual stimulus is presented to the subjects and they passively watch the computer screen. In this study, we aimed to carry out a 6-class classification using the 5F and NoMT paradigms. Whilst recording of EEG signals, the action signal remained on the screen for 1 s to implement the corresponding motor imageries. At the end of the given time, the task was not shown on the screen. Instead, the relevant task was interrupted for 1.5–2.5 s until the next task. In this dataset, two different sampling frequencies, 200 and 1,000 Hz, were set for experiments. EEG signals recorded with a 1000 Hz sampling frequency were extracted to be used in this study. In recording of EEG signals acquired at 1,000 Hz, a 0.53–100 Hz band-pass filter was applied to signals using hardware filters. In addition, a 50 Hz notch filter was applied to reduce the electrical grid interface. Before performing the feature extraction and the following steps, to have a balanced distribution among the classes and provide adjusted chance level (Galiotta et al., 2022), 100 samples of 1,000 Hz EEG signals for the 5F (five classes) and NoMT (one class) paradigms were studied for each class as the preprocessing stage. Hence, a total of 600 trials were performed for one subject. After obtaining the 5F and NoMT EEG signals for each subject, each EEG segment is decomposed to the finite number of PRCs by applying ITD.

### 2.2 Intrinsic time-scale decomposition (ITD)

ITD is introduced by Frei and Osorio for time-frequency-energy (TFE) analysis of signals with precision (Frei and Osorio, 2007). The ITD decomposes a signal into (*i*) a sum of PRCs, and (*ii*) a monotonic trend without the need for laborious and ineffective sifting or splines. It is an iterative decomposition algorithm for the analysis of nonlinear and non-stationary signals, decomposing the original signal into low-frequency, which is known as baseline signal (*L*_*t*_), and high-frequency, which are known as proper rotation (*H*_*t*_) components. ITD preserves precise temporal information (Frei and Osorio, 2007; Voznesensky and Kaplun, 2019; Degirmenci and Akan, 2020).

For the application of ITD, suppose there is an EEG signal *X*_*t*_ to be processed. To extract the low-frequency component (“baseline signal”) from the EEG signal, an operator 𝔏 is introduced and the remainder is the high-frequency component (“proper rotation”). Hence, the EEG signal *X*_*t*_ is defined as in Equation 1.


(1) $$\begin{array}{l} X_{t}=𝔏X_{t}+\left(1-𝔏\right)X_{t}=L_{t}+H_{t} \end{array}$$

where the baseline signal is indicated as *L*_*t*_ = 𝔏*X*_*t*_, and the proper rotation component is indicated as *H*_*t*_ = (1 − 𝔏)*X*_*t*_. The extraction of baseline and proper rotation components are explained in detail with the following three steps (Frei and Osorio, 2007; Martis et al., 2013; Voznesensky and Kaplun, 2019):

- A real-valued signal is assumed as *X*_*t*_, *t* ≥ 0 and τ_*k*_, *k* = 1, 2, ⋯    denotes the its local extremes. Let the value of the signal at τ_*k*_ is denoted as *X*(τ_*k*_) and the value of its baseline at τ_*k*_ is denoted as *L*(τ_*k*_).
- We assume that *L*_*t*_, and *H*_*t*_ have been defined over the interval [0, τ_*k*_], and *X*_*t*_ is available for [0, τ_*k*_ + 2]. The baseline extraction operator, 𝔏 is provided as a piece-wise linear function on the interval (τ_*k*_, τ_*k*_ + 1] between the two extrema as defined in Equations 2, 3.
  (2) $$\begin{array}{l} L_{t}=L_{k}+\left(\frac{L_{k+1}-L_{k}}{L_{k+2}-L_{k}}\right)\left(X_{t}-X_{k}\right),\;t\epsilon \left(\tau_{k},\tau_{k}+1\right] \end{array}$$
  where
  (3) $$\begin{array}{l} L_{k+1}=\alpha \left[X_{k}+\left(\frac{\tau_{k+1}-\tau_{k}}{\tau_{k+2}-\tau_{k}}\right)\left(X_{k+2}-X_{k}\right)\right]+\left(1-\alpha \right)X_{k+1}, \end{array}$$
  and 0 < α < 1, is typically set with $\alpha =\frac{1}{2}$. The baseline signal, *L*_*t*_ is constructed in this way to obtain the monotonicity of *X*_*t*_ between extrema. Hence, the baseline signal is reconstructed as a linearly transformed contraction of the original signal in conformity with Equations 2, 3.
- Once the baseline signal is defined, the residual or high-frequency component, PRC is computed as defined in Equation 4.
  (4) $$\begin{array}{l} HX_{t}=\left(1-𝔏\right)X_{t}=H_{t}=X_{t}-L_{t} \end{array}$$

Using the baseline *L*_*t*_, and the high frequency *H*_*t*_ modes, the original signal *X*_*t*_ can be reconstructed using Equation 5.


(5) $$\begin{array}{l} X_{t}=L_{t}^{D}+\overset{D}{\underset{j=0}{\sum }}H_{t}^{j},\;j=0,\cdots \;,D \end{array}$$

where *D* denotes the number of PRCs that are provided during ITD processing.

**Figure 2:**
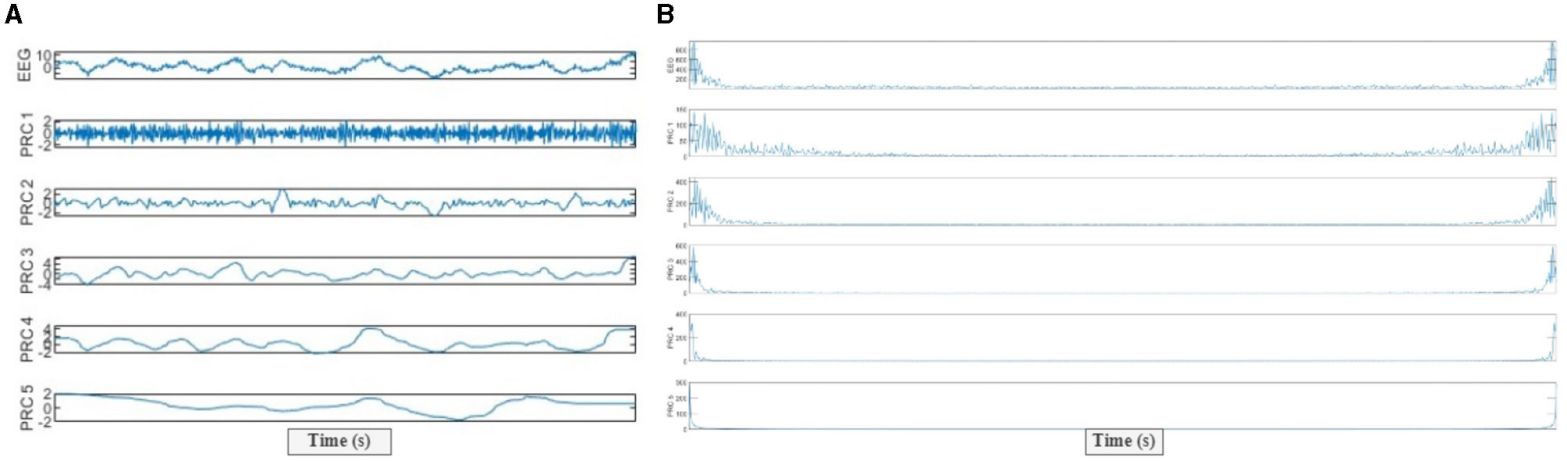
**(A)** PRCs extracted by the intrinsic time-scale decomposition (ITD) from a 1-s segment of EEG signals, **(B)** Energies of each PRCs (the first five modes are given as examples).

An exemplary motor imagery EEG signal decomposition process conducted through the ITD algorithm is given in Figure 2A. To decide which of the separate PRCs to work with, the PRCs were examined in the frequency domain and their energy spectrums were computed. In Figure 2B, a case of energy spectrums of PRCs, decomposed into an EEG signal is provided. Figure 2B shows that the first PRC (i.e., PRC1) has the highest frequency content, while the fifth PRC (i.e., PRC5) exhibits the lowest frequency content. Hence, we selected the first three PRCs and their different combinations for our suggested feature extraction process due to the fact that they include high-frequency contents that best represent the signal characteristic of the original EEG. Various feature extraction methods are implemented to the determined high-frequency PRCs (*H*_*t*_), which are decomposed through ITD. In our study design, seven different sets of high-frequency PRCs which are only PRC1, PRC2, and PRC3, and their different combinations [PRC1–PRC2, PRC1–PRC3, PRC2–PRC3, and PRC1–PRC2–PRC3 (denoted as PRC1-to-3)] are acquired and utilized to evaluate 10 features.

### 2.3 ITD features

Following the extraction of low-frequency baseline signal and high-frequency PRCs by running the ITD algorithm, EEG signal properties including the power, mean value, sample entropy, high-frequency moments (first moment, second moment, third moment, and fourth moment), and Hjorth parameters (activity, mobility, and complexity) were computed from various combinations of PRCs. Their details are described below:

- The **mean value** was calculated based on time-domain information for 3 PRCs. It is defined as in Equation 6.
  (6) $$\begin{array}{l} \mu =\frac{1}{N}\overset{N-1}{\underset{n=0}{\sum }}X\left[n\right] \end{array}$$
  where PRCs are denoted as *X*[*k*], the mean value is denoted as μ and the size of PRCs is described as *N*.
- The **total power** of PRCs was obtained using the spectrum of signals. The spectrum of PRCs was evaluated by implementing the periodogram method, which allows for analysis of the frequency content of a signal (Iscan et al., 2011; Karabiber Cura et al., 2023). From definitions of *k*-th frequency (Equation 7) and power power spectral desity estimation of the *k*-th frequency component (Equation 8), the total power is defined as in Equation 9 (Iscan et al., 2011):
  (7) $$\begin{array}{l} w_{k}=\frac{2\pi }{N}k,k=0,1,\cdots \;,N-1 \end{array}$$
  (8) $$\begin{array}{l} S\left(w_{k}\right)=\frac{1}{N}|X\left(w_{k}\right){|}^{2} \end{array}$$
  (9) $$\begin{array}{l} S_{T}=\overset{N-1}{\underset{k=0}{\sum }}S\left(w_{k}\right) \end{array}$$
  where *S*(*w*_*k*_) indicates the power spectral density of the signal provided by the periodogram method, *X*(*w*_*k*_) indicates the discrete Fourier transform of the PRC *x*[*n*], and *S*_*T*_ is the total power of PRCs. *N* shown in Equations 8, 9, refers to the size of the corresponding signal.
- The **higher order spectral moments (1st, 2nd, 3rd, and 4th)** were computed using the spectrum of signals like total power. These moments are defined as in Equations 10–13, respectively (Degirmenci et al., 2018):
  (10) $$\begin{array}{l} M_{1}=\overset{N-1}{\underset{k=0}{\sum }}{\left(w_{k}\right)}^{1}S\left(w_{k}\right) \end{array}$$
  (11) $$\begin{array}{l} M_{2}=\overset{N-1}{\underset{k=0}{\sum }}{\left(w_{k}\right)}^{2}S\left(w_{k}\right) \end{array}$$
  (12) $$\begin{array}{l} M_{3}=\overset{N-1}{\underset{k=0}{\sum }}{\left(w_{k}\right)}^{3}S\left(w_{k}\right) \end{array}$$
  (13) $$\begin{array}{l} M_{4}=\overset{N-1}{\underset{k=0}{\sum }}{\left(w_{k}\right)}^{4}S\left(w_{k}\right) \end{array}$$
  Here, *M*_1_, *M*_2_, *M*_3_, and *M*_4_ represent the 1st, 2nd, 3rd, and 4th higher order spectral moments of the corresponding PRCs, respectively.
- **Hjorth parameters** were introduced by Hjorth (1970) in 1970, and these are time-domain statistical features used in signal processing. These parameters include the **Activity parameter (*A*_*x*_), Mobility parameter (*M*_*x*_), and Complexity parameter (*C*_*x*_)** of the signal. In the following mathematical equations for Activity, Mobility, and Complexity parameters, *y*(*n*) indicates the auto-correlation function of one PRC after the ITD application. *y*[*n*] = [*y*1, *y*2, ⋯   , *yN*], and *N* indicates the length of the signal. **Activity parameter**, defines the power of vibration signal and can be evaluated using the variance of signal amplitude. It is formulated in Equation 14 (Hjorth, 1970; Yu and Fang, 2022):
  (14) $$\begin{array}{l} A_{x}=\left(y\left(n\right)\right)=\sigma_{y}^{2} \end{array}$$
  where σ_*y*_ denotes the standard deviation of *y*(*n*) and it can be described with the Equation 15.
  (15) $$\begin{array}{l} \sigma_{y}=\sqrt{\frac{1}{N-1}\overset{N}{\underset{n=1}{\sum }}{\left[y\left(n\right)-\mu \right]}^{2}} \end{array}$$
  Here, the mean value of the signal is represented with μ. **Mobility parameter** describes the ratio of standard deviations of first-order derivatives, and it can be evaluated using the slope of the signal. It is defined as in Equation 16.
  (16) $$\begin{array}{l} M_{x}=\sqrt{\frac{\sigma_{y^{\prime }}^{2}}{\sigma_{y}^{2}}}=\frac{\sigma_{y^{\prime }}}{\sigma_{y}} \end{array}$$
  where $\sigma_{y^{\prime }}$ indicates the first-order standard deviation of signals. **Complexity parameter** denotes the similarity of signal to sinusoidal signal and it is expressed as the ratio between the mobility of the first derivative of the EEG signal and the mobility of the EEG signal itself (Hjorth, 1970; Yu and Fang, 2022). The mathematical expression of complexity parameters is given in Equation 17.
  (17) $$\begin{array}{l} C_{x}=\frac{M_{x}\left(y^{\prime }\left(t\right)\right)}{M_{x}\left(y\left(t\right)\right)}=\frac{M_{x}\left(\frac{dy\left(t\right)}{dt}\right)}{M_{x}\left(y\left(t\right)\right)}=\sqrt{\frac{\frac{\sigma_{y^{\prime \prime }}^{2}}{\sigma_{y^{\prime }}^{2}}}{\frac{\sigma_{y^{\prime }}^{2}}{\sigma_{y}^{2}}}} \end{array}$$
  Here, the second-order standard deviation of signal *y*(*t*) is expressed as $\sigma_{y^{\prime \prime }}$.
- The **sample entropy** indicates a time series complexity measure that represents the probability of a system generating new patterns. It can be defined as the embedding theory that utilizes the time series directly instead of probability values. The original time series is defined as *L*_*t*_(*i*), *i* = 1, 2, ⋯   , *N*. The new vector sequences which each of size *m*, *u*(1) by *u*(*N* − *m* + 1) are created, and expressed as *u*(*i*) = {*L*_*t*_(*i*), *L*_*t*_(*i* + 1), ⋯   , *L*_*t*_(*i* + *m* − 1)} (Higuchi, 1988; Martis et al., 2013). The defined length *m* indicates the embedding dimension. The distance *d*[*u*(*i*), *u*(*j*)] between vectors *u*(*i*), and *u*(*j*) is described in Equation 18 (Higuchi, 1988):
  (18) $$\begin{array}{l} d\left(u\left(i\right),u\left(j\right)\right)=max\left\{\left|u\left(i+k\right)-u\left(j+k\right)\right|\right\},0\leq k\leq m-1 \end{array}$$
  Here, *k* is an index. The probability of providing another vector within a distance *r* from vector *u*(*i*) is defined as in Equation 19 (Higuchi, 1988):
  (19) $$\begin{array}{l} C_{i}^{\prime m}\left(r\right)=\frac{1}{N-m+1} \end{array}$$
  The number of j, *j* ≠ *i, j* ≤ *N* − *m* + 1 such that *d*(*u*(*i*), *u*(*j*)) ≤ *r* The entropy can be defined in Equation 20.
  (20) $$\begin{array}{l} \emptyset^{m}\left(r\right)={\left(N-m+1\right)}^{-1}\overset{N-m+1}{\underset{i=1}{\sum }}C_{i}^{\prime m}\left(r\right) \end{array}$$
  Then, the sample entropy is described in Equation 21 (Martis et al., 2013):
  (21) $$\begin{array}{l} SampEn\left(m,r,N\right)=-ln\left[\frac{\emptyset^{\prime m}\left(r\right)}{\emptyset^{\prime m+1}\left(r\right)}\right] \end{array}$$

### 2.4 Feature reduction using statistical significance (ANOVA)

Applying too many features to classifiers could unnecessarily complicate the implementation of classifiers. The application of redundant information in EEG signals can cause confusion, which is defined as the curse of dimensionality (Hart et al., 2000). Trying different combinations one by one and finding the most suitable classification causes computational load (Narin et al., 2014). Feature reduction algorithms can be used instead of feature selection based on trying different combinations. The purpose of feature reduction is to investigate small-size subsets of features that can provide the same or better optimal classification performances (Yesilkaya et al., 2023). Using fewer data presenting some relevant features of motor imagery EEG signals is important to obtain optimal classifier performance without computational load.

In this study, a feature reduction method based on statistical significance was applied to determine relevant ITD features that provide the best discrimination of the FM imageries for each sample. The statistical significance-based feature selection method used in this study was also performed in other BCI studies (Bulut et al., 2022; Degirmenci et al., 2022c, 2023). One-way variance analysis (ANOVA test), which is mainly used to indicate whether there is a difference between the means in conditions where there are two or more groups was used in this study. We preferred the ANOVA test from statistical significance-based feature selection methods since a total of six motor imagery tasks including five FM imageries and NoMT cases tried to be classified. Thus, the effect of the ANOVA test-based feature selection method was investigated with ITD features. The statistical significance of all extracted EEG features was determined by calculating *p*-values. The statistical significance level (α) is defined as 0.05 and the features that ensure the statistical evidence range were indicated and selected as statistically significant features. In addition to the classifications performed without the feature selection process in our study, the feature vector including selected statistically significant ITD features were also given to the classification algorithms as input data to differentiate FM imageries. The effectiveness of the ANOVA-based feature selection process is investigated by comparing the results of classifications with all features and selecting statistically significant features.

### 2.5 Classification

In this study for differentiation of FM imageries, the provided ITD-based EEG features have been evaluated using eight well-known machine learning algorithms, such as Decision Tree (Tzallas et al., 2009; Sharma et al., 2022), Discriminant Analysis (Hart et al., 2000; Chakrabarti et al., 2003; Lotte et al., 2018), Naive Bayes (Hart et al., 2000; Miao et al., 2017), Support Vector Machine (Vapnik, 1999; Hart et al., 2000; Bascil et al., 2016), *k*-Nearest Neighbor (Hart et al., 2000; Isler, 2009; Tzallas et al., 2009), Ensemble Learning (Sayilgan et al., 2019, 2020, 2021a,b, 2022; Degirmenci et al., 2022b,c; Karabiber Cura et al., 2023), Neural Networks (Richard and Lippmann, 1991; Pan et al., 2012; Narin and Isler, 2021; Ozdemir et al., 2021; Degirmenci et al., 2022a), and Kernel Approximation (Maji et al., 2008; Lei et al., 2019). The classifiers and corresponding algorithms that were adopted in this study are listed below in Table 1. Each of these algorithms was implemented via utilizing the Classification Learner Toolbox, which is part of the Statistics and Machine Learning Toolbox available in the Matlab software package (Matlab, 2023). Since the technical details of these classifiers have become so trivial that inherited details are not explained. For further details regarding the classifiers, studies that are cited in the table can be accessed.

**Table 1:** List of adopted classifiers with their implemented algorithms.

**Classifier**	**Algorithms**
Decision Tree	Fine, medium, and coarse
Discriminant Analysis	Linear, and quadratic
Naive Bayes	Gaussian, kernel
Support Vector Machine (SVM)	Linear, quadratic, cubic, fine Gaussian, medium Gaussian, coarse Gaussian
*k*-Nearest Neighbor (kNN)	Cubic, cosine
Ensemble Learning	Boosted, Bagged, Subspace Discriminant, Subspace *k*-NN, RUSBoosted Trees
Neural Networks	Narrow, medium, wide, bi-layered, tri-layered
Kernel Approximation	Support vector machine, logistic regression

### 2.6 Performance evaluation

Training is defined as updating the classifier-specific parameters according to the available data. Testing is determining the performance of classifiers by the correct decisions made on the unseen data before. For this reason, the feature set was divided into two groups as train data (80%) and test data (20%) using the random splitting method (Hart et al., 2000).

In addition, during training, classifiers are expected to generalize rather than over-fit (or memorize) the available data. However, it may be difficult to make generalizations, especially when the size of the data is not large enough. Cross-validation (CV) is a method employed to evaluate the predictive performance of a model on data it has not processed (classified) before. Several cross-validation methods, including hold-out, leave-one-out, *k*-fold, and Monte-Carlo (MC) exist. All in all, hold-out (*k* equals 2) and leave-one-out (*k* equals the number of samples) methods are special cases of the *k*-fold method (Hart et al., 2000; Isler et al., 2015; Patro, 2021).

Differences between *k*-fold and MC methods are emphasized in the recent literature: (a) the *k*-fold uses each data in the validation although MC uses samples arbitrary times (0 or more), (b) the *k*-fold divides the data into k parts, although MC separates large number data parts, (c) the *k*-fold results in unbiased accuracy with a high variance where the MC results in highly biased accuracy with low variance. These differences cause a trade-off among CV methods (Patro, 2021). A recent study emphasizes that a large number of simulated data may cause over-fitting and using independent data for extra validation is necessary (Labriffe et al., 2022).

Therefore, we preferred the *k*-fold CV method as in our similar studies (Isler, 2009; Isler and Kuntalp, 2009; Degirmenci et al., 2022a,b) and the recent literature (Anam et al., 2019, 2020; Kato et al., 2020; Mwata-Velu et al., 2021, 2022; Azizah et al., 2022; Zahra et al., 2022). Using *k*-fold cross-validation (CV), the training data set was divided into *k* equal-sized subsets. One subset was used as test data, other subsets (*k* − 1) were determined as training data, and this classification process was repeated *k* times (Hart et al., 2000). Regarding Brownlee's article on the Machine Learning Mastery website (Brownlee, 2023), there is no general rule for choosing the k value, but as the k value decreases, the bias value also decreases (Kuhn and Johnson, 2013). Additionally, it is stated that empirically selected values of 5 or 10 give a balanced bias-variance test error (James et al., 2013). The average classification performance of these iterations is defined as the training performance (Hart et al., 2000). In conclusion, *k* was set as 5 for this study as in similar studies.

The accuracy (ACC) performance criterion is used in this study to evaluate the performance of various machine learning algorithms. The mathematical expression of the accuracy performance criterion is given in Equation 22 (Hart et al., 2000).


(22) $$\begin{array}{l} ACC=\frac{TP+TN}{TP+FN+TN+FP} \end{array}$$

Here, *TP* and *TN* indicate the number of correctly assigned samples into the true class. In addition, *FP* and *FN* indicate the number of incorrectly assigned samples into positive class and negative class, respectively.

## 3 Results

The suggested methods were applied to EEG segments of 19-channel EEG signals collected from 8 subjects. Firstly, the ITD approach was used to decompose EEG signals into PRCs. Then the power, mean value, sample entropy, high-frequency moments (first moment, second moment, third moment, and fourth moment), and Hjorth parameters (activity, mobility, and complexity) were evaluated as features utilizing distinct combinations of PRCs. In the feature extraction process performed in this study, both the first three components (PRC1, PRC2, and PRC3) and their different combinations (PRCs1-2, PRCs1-3, PRCs2-3 and PRC1-to-3) were used and their effectiveness was investigated, individually. The same feature extraction process was also performed on EEG signals without any ITD approach to show the effectiveness of the ITD algorithm in FM classification. Additionally, the ANOVA-based feature selection process was carried out on the PRC1-to-3 feature set and its effectiveness was investigated. Finally, a variety of classifiers including Decision Tree, Discriminant Analysis, Naive Bayes, Support Vector Machine, *k*-Nearest Neighbor, Ensemble Learning, Neural Networks, and Kernel Approximation were used to classify FM imagery of EEG segments, and the experimental results of each were analyzed.

The classification performances of the ITD-based features computed using the different components and EEG-based features were evaluated to compare and analyze the effectiveness of the suggested ITD-based process. The classification performances of features acquired through our suggested ITD-based approaches with various classifiers are given in Tables 2–**9**. These classification performances were evaluated using both feature sets provided using single PRCs (PRC1, PRC2, and PRC3), their combinations (PRCs1-2, PRCs1-3, and PRC1-to-3), and ANOVA-selected PRC1-to-3 combination. In tables, EEG indicates that the feature set utilized in the classification step is generated using the EEG signal itself without applying ITD. Additionally, boldface characters show which feature set obtained the highest accuracy performance in subject-dependent and subject-independent analyses separately.

**Table 2:** All components' performances were tested in this study using the Decision Tree classifier.

**Components**	**S1**	**S2**	**S3**	**S4**	**S5**	**S6**	**S7**	**S8**	**SI**
PRC1	29.17	27.50	35.00	32.50	**29.17**	25.83	31.67	27.50	**25.83**
PRC2	26.67	28.33	26.67	30.00	26.67	**30.83**	30.00	**32.50**	20.63
PRC3	24.17	28.33	26.67	34.17	**29.17**	20.83	28.33	27.50	21.77
PRCs1-2	26.67	26.67	26.67	37.50	27.50	29.17	32.50	30.00	23.13
PRCs1-3	26.67	**30.83**	30.00	34.17	27.50	29.17	29.17	30.00	24.79
PRCs2-3	**35.83**	26.67	26.67	35.83	25.00	25.83	23.33	24.17	22.71
PRCs1-to-3	27.50	30.00	28.33	36.67	26.67	27.50	**36.67**	22.50	23.54
ANOVA+PRCs1-to-3	30.83	30.00	**35.83**	**44.17**	26.67	27.50	34.17	27.50	24.06
EEG	30.00	26.67	32.50	35.83	25.00	30.00	25.83	27.50	25.31
ANOVA+EEG	23.33	**30.83**	35.00	38.33	21.67	30.00	25.00	30.83	23.33

The maximum component accuracies are shown in boldface for each subject where SI means (subject-independent).

Decision Tree classification performances evaluated using ITD-based features are presented in Table 2. With respect to these results, the first three PRCs combined with an ANOVA-based feature selection process obtain the highest accuracy value of 44.17% in S4 (Subject E). The performance comparison of the ITD-based approach with the EEG-based case (without the ITD process), shows that the highest performance values were obtained with the use of ITD-based features in all subjects except S2 (Subject B). The results for S2 (Subject B) were further investigated and it was noticed that the highest accuracy value reported was 30.83% in classifications performed using both PRCs1-3 combination and EEG features with an ANOVA-based feature selection process.

Linear Discriminant Analysis classification performances were evaluated using ITD-based features presented in Table 3. When the results are compared, the first three PRCs combined with the ANOVA-based feature selection process obtain the highest accuracy value of 47.50% in S4 (Subject E). The comparison of classifier performances with the features extracted through the ITD-based approach and the performances of the same classifiers with the features of the EEG-based case (without the ITD process) could not be conducted clearly since the results of the EEG-based case could not be computed. The EEG-based feature set could not be classified because they do not fit the Linear Discriminant Analysis classifier's parameters.

**Table 3:** All components' performances were tested in this study using the Linear Discriminant Analysis classifier.

**Components**	**S1**	**S2**	**S3**	**S4**	**S5**	**S6**	**S7**	**S8**	**SI**
PRC1	25.83	28.33	33.33	27.50	24.17	30.83	27.50	26.67	26.25
PRC2	24.17	28.33	30.00	40.00	24.17	30.83	27.50	27.50	24.79
PRC3	27.50	25.00	32.50	27.50	31.67	**31.67**	21.67	25.83	29.17
PRCs1-2	31.67	25.83	35.00	34.17	27.50	26.67	21.67	22.50	29.38
PRCs1-3	31.67	26.67	**38.33**	43.33	**35.83**	30.83	25.00	29.17	29.90
PRCs2-3	32.50	25.83	28.33	27.50	25.83	25.83	**32.50**	25.83	28.85
PRCs1-to-3	31.67	25.00	26.67	33.33	29.17	25.00	20.83	25.00	30.83
ANOVA+PRCs1-to-3	**38.33**	**40.00**	37.50	**47.50**	**35.83**	28.33	28.33	**30.00**	**33.54**
EEG	N/A	N/A	N/A	N/A	N/A	N/A	N/A	N/A	N/A
ANOVA+EEG	N/A	N/A	N/A	N/A	N/A	N/A	N/A	N/A	N/A

The maximum component accuracies are shown in boldface for each subject where SI means (subject-independent).

Naive Bayes classification performances evaluated using ITD-based features are presented in Table 4. According to these results, EEG features with an ANOVA-based feature selection process obtain the highest accuracy value of 40.00% in S4 (Subject E). The performances of the ITD-based approach were compared with the performances of the EEG-based case (without the ITD process), and the comparison reflects that the highest performance values were obtained with the use of ITD-based features in all subjects except three subjects. The analyses performed for S3 (Subject C) were further investigated, it was found that the highest accuracy value was 34.17% in classifications performed using both the first three PRCs combination with ANOVA-based feature selection and EEG features with ANOVA-based feature selection process.

**Table 4:** All components' performances were tested in this study using the Naive Bayes classifier.

**Components**	**S1**	**S2**	**S3**	**S4**	**S5**	**S6**	**S7**	**S8**	**SI**
PRC1	22.50	30.83	32.50	35.00	25.00	27.50	30.00	31.67	20.94
PRC2	27.50	25.00	29.17	34.17	24.17	24.17	24.17	25.83	19.38
PRC3	26.67	22.50	25.00	32.50	30.83	25.00	23.33	**32.50**	20.31
PRCs1-2	24.17	26.67	29.17	30.83	27.50	22.50	**30.83**	25.00	22.19
PRCs1-3	26.67	26.67	33.33	37.50	**38.33**	30.00	29.17	23.33	20.94
PRCs2-3	29.17	25.00	30.83	30.00	31.67	25.00	21.67	27.50	21.15
PRCs1-to-3	23.33	30.00	30.83	35.83	23.33	27.50	23.33	27.50	23.96
ANOVA+PRCs1-to-3	30.83	**35.00**	**34.17**	39.17	31.67	**35.83**	**30.83**	30.83	22.81
EEG	31.67	30.83	28.33	40.83	26.67	22.50	20.83	21.67	**25.42**
ANOVA+EEG	**32.50**	25.83	**34.17**	**40.00**	27.50	29.17	19.17	21.67	23.23

The maximum component accuracies are shown in boldface for each subject where SI means (subject-independent).

Support Vector Machine classification performances evaluated using ITD-based features are presented in Table 5. The results expose that the first three PRCs combined with an ANOVA-based feature selection process and without an ANOVA-based feature selection process obtain the highest accuracy value of 49.17% in S4 (Subject E). On the other hand, the same highest accuracy value is also found for the first three PRCs in combination with the ANOVA-based feature selection process in S3 (Subject C). The performances of the ITD-based approach were compared with the performances of the EEG-based case (without the ITD process) and the comparison shows that the highest performance values were obtained with the use of ITD-based features in all subjects.

**Table 5:** All components' performances were tested in this study using the Support Vector Machine classifier.

**Components**	**S1**	**S2**	**S3**	**S4**	**S5**	**S6**	**S7**	**S8**	**SI**
PRC1	29.17	35.83	40.83	40.00	30.00	**38.33**	**40.00**	32.50	30.00
PRC2	22.50	25.83	34.17	35.00	30.00	35.83	31.67	24.17	25.73
PRC3	31.67	29.17	30.00	40.00	33.33	29.17	28.33	26.67	27.08
PRCs1-2	31.67	31.67	44.17	40.00	24.17	33.33	39.17	27.50	30.52
PRCs1-3	35.83	38.33	41.67	47.50	**38.33**	35.00	31.67	29.17	32.19
PRCs2-3	29.17	32.50	39.17	37.50	**38.33**	30.83	35.00	32.50	28.13
PRCs1-to-3	27.50	37.50	45.00	**49.17**	33.33	**38.33**	35.00	35.83	30.63
ANOVA+PRCs1-to-3	**40.00**	**45.00**	**49.17**	**49.17**	35.83	36.67	39.17	**36.67**	**34.48**
EEG	30.00	41.67	38.33	45.00	32.50	33.33	29.17	29.17	31.46
ANOVA+EEG	27.50	41.67	43.33	47.50	34.17	33.33	30.00	29.17	33.65

The maximum component accuracies are shown in boldface for each subject where SI means (subject-independent).

*k*-Nearest Neighbors classification performances acquired using ITD-based features are presented in Table 6. According to these results, the PRCs1-3 combination obtains the highest accuracy value of 46.67% in S3 (Subject C). The performances of the ITD-based approach were compared with the performances of the EEG-based case (without the ITD process) and the highest performance values were obtained with the use of ITD-based features in all subjects.

**Table 6:** All components' performances were tested in this study using the *k*-Nearest Neighbors classifier.

**Components**	**S1**	**S2**	**S3**	**S4**	**S5**	**S6**	**S7**	**S8**	**SI**
PRC1	24.17	38.33	35.00	35.83	29.17	34.17	32.50	**37.50**	29.90
PRC2	23.33	25.00	26.67	33.33	31.67	30.00	28.33	23.33	23.02
PRC3	33.33	25.00	34.17	38.33	28.33	30.00	25.83	30.00	26.88
PRCs1-2	32.50	30.83	34.17	36.67	26.67	32.50	30.83	30.83	28.54
PRCs1-3	31.67	29.17	39.17	**46.67**	32.50	34.17	29.17	30.83	29.48
PRCs2-3	32.50	26.67	32.50	35.83	32.50	**35.00**	28.33	26.67	26.25
PRCs1-to-3	28.33	30.83	35.00	44.17	25.83	31.67	30.83	32.50	26.88
ANOVA+PRCs1-to-3	**35.83**	**39.17**	**43.33**	45.83	**35.00**	34.17	**36.67**	35.83	**30.00**
EEG	30.00	33.33	33.33	43.33	26.67	29.17	31.67	30.00	27.81
ANOVA+EEG	30.00	33.33	40.83	40.00	31.67	31.67	29.17	32.50	28.64

The maximum component accuracies are shown in boldface for each subject where SI means (subject-independent).

Ensemble Learning classification performances evaluated using ITD-based features are presented in Table 7. With regard to these results, the first three PRCs combined with an ANOVA-based feature selection process obtained the highest accuracy value of 55.00% for S4 (Subject E). When the performances of the ITD-based approach were compared with the performances of the EEG-based case (without the ITD process), it was evident that the highest performance values were obtained with the use of ITD-based features in all subjects.

**Table 7:** All components' performances were tested in this study using the Ensemble Learning classifier.

**Components**	**S1**	**S2**	**S3**	**S4**	**S5**	**S6**	**S7**	**S8**	**SI**
PRC1	29.17	32.50	41.67	35.83	29.17	35.00	37.50	34.17	29.69
PRC2	30.83	30.00	36.67	38.33	28.33	31.67	29.17	30.00	25.10
PRC3	29.17	32.50	34.17	41.67	27.50	32.50	27.50	29.17	26.46
PRCs1-2	32.50	34.17	40.00	41.67	33.33	29.17	33.33	32.50	28.85
PRCs1-3	35.83	36.67	40.00	43.33	34.17	34.17	38.33	35.83	31.56
PRCs2-3	**36.67**	29.17	37.50	45.00	31.67	30.00	28.33	30.83	29.06
PRCs1-to-3	34.17	35.00	40.83	47.50	32.50	30.83	36.67	31.67	32.08
ANOVA+PRCs1-to-3	35.83	**40.83**	**50.83**	**55.00**	**37.50**	**36.70**	**41.67**	**39.17**	**32.60**
EEG	30.83	40.00	43.33	39.17	35.83	36.67	26.67	31.67	29.06
ANOVA+EEG	29.17	38.33	45.83	39.17	35.00	35.00	27.50	35.00	27.60

The maximum component accuracies are shown in boldface for each subject where SI means (subject-independent).

Neural Networks classification performances evaluated using ITD-based features are presented in Table 8. The results indicate that the first three PRCs combined with an ANOVA-based feature selection process achieved the highest accuracy value of 53.00% for S3 (Subject C). Comparison of the performances of the ITD-based approach with the performances of the EEG-based case (without the ITD process) shows that the highest performance values were realized with the use of ITD-based features in all subjects except S6 (Subject G) and S8 (Subject I). Further analyses performed for S6 (Subject G) showed that the highest accuracy value attained was 38.33% in classifications performed using both the first three PRCs' combination with ANOVA-based feature selection process and EEG features with ANOVA-based feature selection process. On the other hand, the analyses performed for S8 (Subject I) revealed that the highest accuracy value reached was 35.00% using EEG features with an ANOVA-based feature selection process.

**Table 8:** All components' performances were tested in this study using the Neural Networks classifier.

**Components**	**S1**	**S2**	**S3**	**S4**	**S5**	**S6**	**S7**	**S8**	**SI**
PRC1	33.33	29.17	30.83	39.17	23.33	33.33	31.67	29.17	26.25
PRC2	25.00	25.00	31.67	31.67	25.83	25.83	25.83	30.00	24.48
PRC3	32.50	20.83	35.83	35.00	30.00	25.83	32.50	28.33	25.94
PRCs1-2	27.50	30.00	43.33	42.50	33.33	30.83	27.50	26.67	28.75
PRCs1-3	**34.17**	32.50	40.83	42.50	35.00	30.00	31.67	31.67	30.94
PRCs2-3	29.17	29.17	37.50	35.00	34.17	35.83	31.67	30.83	28.96
PRCs1-to-3	30.00	33.33	45.83	**48.33**	**37.50**	32.50	30.00	34.17	29.27
ANOVA+PRCs1-to-3	**34.17**	**42.50**	**53.33**	45.83	**37.50**	**38.33**	**35.00**	31.67	**31.88**
EEG	28.33	35.83	42.50	39.17	35.00	29.17	26.67	32.50	28.96
ANOVA+EEG	25.83	35.00	41.67	42.50	36.67	**38.33**	23.33	**35.00**	30.42

The maximum component accuracies are shown in boldface for each subject where SI means (subject-independent).

Kernel Approximation classification performances evaluated using ITD-based features are presented in Table 9. In reference to the results, one can infer that the first three PRCs combination without an ANOVA-based feature selection process obtained the highest accuracy value of 40.83% in S4 (Subject E). The performances of the ITD-based approach and the performances of the EEG-based case (without the ITD process) were compared and it was apparent that the highest performance values were obtained with the use of ITD-based features in only S4 (Subject E) and S7 (Subject H). In S1 (Subject A), S3 (Subject C), S6 (Subject G), and S8 (Subject I), the highest performance values were obtained with the use of EEG-based features with or without an ANOVA-based feature selection process. In other subjects, the highest performance values were obtained with the use of both ITD-based features and EEG-based features.

**Table 9:** All components' performances were tested in this study using the Kernel Approximation classifier.

**Components**	**S1**	**S2**	**S3**	**S4**	**S5**	**S6**	**S7**	**S8**	**SI**
PRC1	20.00	**25.00**	25.83	24.17	26.67	25.83	**30.83**	20.83	23.23
PRC2	26.67	**25.00**	23.33	30.00	23.33	17.50	23.33	24.17	19.27
PRC3	27.50	20.00	27.50	35.83	21.67	22.50	21.67	22.50	21.88
PRCs1-2	22.50	21.67	20.83	22.50	19.17	19.17	26.67	25.00	19.58
PRCs1-3	25.83	24.17	27.50	39.17	**27.50**	22.50	29.17	20.00	24.48
PRCs2-3	25.00	20.83	27.50	33.33	20.00	23.33	21.67	19.17	24.27
PRCs1-to-3	24.17	24.17	27.50	**40.83**	15.00	25.83	25.00	25.83	23.23
ANOVA+PRCs1-to-3	21.67	18.33	26.67	31.67	19.17	22.50	22.50	25.83	21.88
EEG	25.83	**25.00**	38.33	32.50	25.00	**30.00**	27.50	**29.17**	24.17
ANOVA+EEG	**29.17**	22.50	**34.17**	36.67	**27.50**	26.67	20.00	26.67	**25.31**

The maximum component accuracies are shown in boldface for each subject where SI means (subject-independent).

## 4 Discussion

The observed results reveal that the ITD algorithm mostly yields a considerable improvement in classification performance when the classification performance of ITD-based approaches are compared with the classification performance of EEG-based analysis conducted without utilizing the ITD algorithm. The highest accuracy values are obtained using the ITD algorithm for most of all classification algorithms except the Naive Bayes algorithm. Among all ITD-based feature sets, all PRCs and their combinations provide a higher classification performance compared to the EEG case in most of the classifications except the Naive Bayes and Kernel Approximation classifications. The classification performance of a single PRC is lower compared to their combinations. The most successful component is the first three PRC combinations (PRC1-to-3). In addition to using PRCs1-to-3, the classification performance is further improved with the implementation of an ANOVA-based feature selection process. The experimental results revealed that the evaluation of different components together provides the highest performance and improves the classification performance.

**Figure 3:**
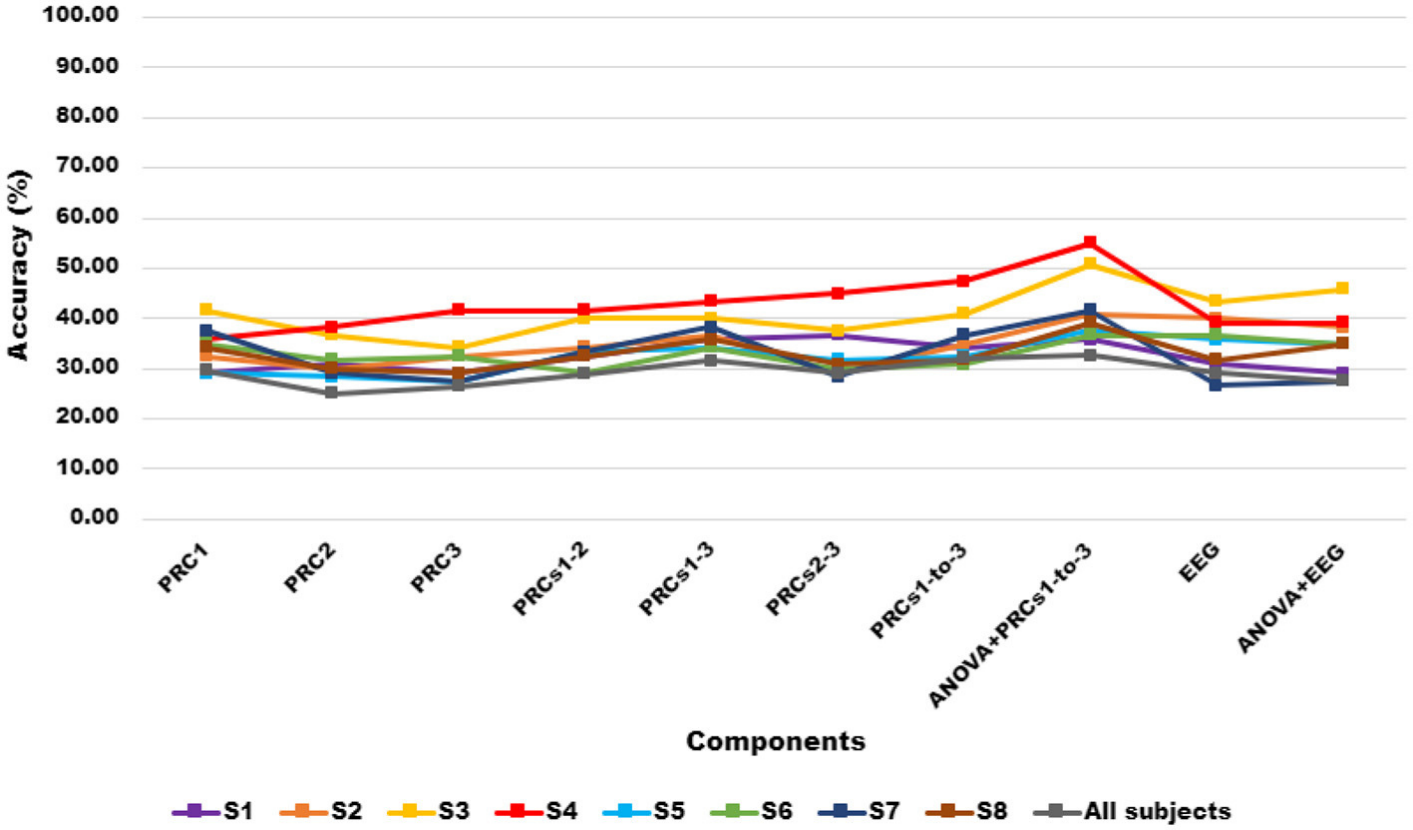
The component-based classification accuracies in Ensemble Learning classifier for all subjects.

Next, the component-based and EEG-based classification accuracies in the Ensemble Learning classifier for subject-dependent and subject-independent cases have been investigated to reveal the efficacy of the proposed ITD-based method more accurately. The performances that are obtained using both feature sets generated utilizing EEGs, single PRCs (PRC1, PRC2, and PRC3), and their combinations (PRCs1-2, PRCs1-3, and PRC1-to-3) by running Ensemble Learning are given in Figure 3. The results reveal that the ITD algorithm provides a significant improvement in terms of accuracy performance compared to the classification performed without using the algorithm. Additionally, the combinations of different components achieved the highest classification performance for subject-dependent and subject-independent cases. Moreover, ANOVA-selected the first three PRC combinations (PRC1-to-3) realized the highest classification performance in analyses for all subjects except S1 (Subject A).

**Figure 4:**
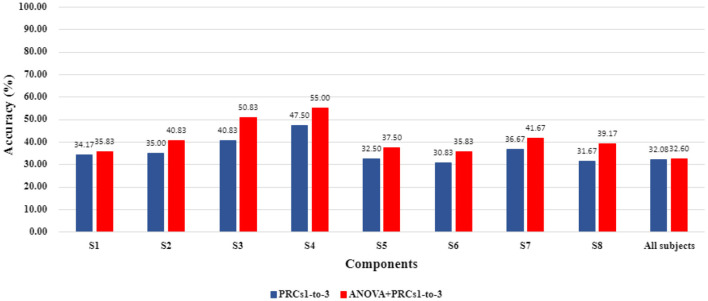
Comparison of accuracy values evaluated using PRCs1-to-3 features and ANOVA-selected PRCs1-to-3 features as regards Ensemble Learning classifier.

The classification performance of ITD-based features from different PRCs with ANOVA-based feature selection and without feature selection process were compared on the basis of providing more accurate information about the performance of the suggested ANOVA-selected ITD features. The classification accuracies for the PRC1-to-3 combination and ANOVA-selected PRC1-to-3 combination achieved by the Ensemble Learning classifier are presented in Figure 4. It can be noticed that the ANOVA-selected PRC1-to-3 combination succeeded in higher classification accuracies than the PRC1-to-3 combination for both subject-dependent and subject-independent cases. The observed results reveal that the suggested statistical significance-based feature reduction process obtains considerably noticeable differences and improves the classifiers' performance.

The results of our study are compared to the state-of-the-art studies, which conducted FM classification based on EEG signals. Table 10 presents a comparison of the suggested study to relevant prior studies. Clearly, both subject-dependent (Kaya et al., 2018; Anam et al., 2019, 2020; Kato et al., 2020; Mwata-Velu et al., 2021, 2022; Azizah et al., 2022) and subject-independent (Kaya et al., 2018; Zahra et al., 2022) studies were conducted for FM classification in literature. In general, the highest performance values were achieved in subject-dependent classification as in our study. An important distinction between studies regarding FM classification was the number of subjects. In some studies (Anam et al., 2019, 2020; Mwata-Velu et al., 2022), classification was computed over the EEG data of four subjects. In contrast, some studies computed and reported using data from eight subjects. As an example of four-subject studies, Anam et al. (2019) reports on the analysis of the data of only four subjects and the classification performance varied between 51.00 and 56.00%. To make a meaningful comparison between the results of Anam et al. (2019) and our study, the sample sizes must be equal. Hence, we think that the two results are incomparable. In Anam et al. (2020), in addition to working with only four subjects, classification was carried out with deep learning structures. Despite the fact that the hidden layers in deep learning structures create a significant amount of workload and necessitate a significant amount of time for training, the reported classification performance in all subjects was not as high as expected (over 90.00%). In another study (Zahra et al., 2022), another deep learning-based classification with very high training time was adopted and considering the same drawbacks of the previous study (Anam et al., 2020), although a significant improvement in performance was achieved since the sample size of this study (i.e., only four subjects) and number of EEG channels (i.e., only four channels) were limited when compared with the sample size and number of EEG channels in our study. Thus, a comparison between the results of this study (Anam et al., 2020) and ours would not be meaningful. On the other hand, some of these prior studies (Mwata-Velu et al., 2021, 2022; Azizah et al., 2022) performed channel reduction. In these studies, four out of all 19 channels were defined as effective channels and used for the feature extraction stage. Among these studies, although deep learning-based classification was performed in addition to channel reduction in the Mwata-Velu et al. (2021), the performance values were only as high as 76%. In one of the studies of the same set (Mwata-Velu et al., 2022), EEG signals of 4 subjects were included, and deep learning-based classification was performed together with the channel reduction process. When their classification results are examined and compared, it is clear that high performances were obtained with regard to already noted certain limitations in the study design. However, our study uses passive condition (NoMT case) EEG signals in addition to EEG signals of FM. Prior studies had focused only on FM and classified them without considering the passive state of the subjects. The 6-class FM classification study we propose appears to be more suitable for the real BCI design and applications. In this study, we used ITD-based features for FM classification. According to our experimental results, 55.00% is the highest accuracy achieved using the pair of the ANOVA-selected first three PRC combinations and the Ensemble Learning classifier.

**Table 10:** Comparison of classifier performances with the state-of-the-art studies for both subject-independent and subject-dependent cases from the literature.

**Study**	***N***	***n***	**Classifier**	**c**	**CV**	**Accuracy (%)**
**Subject-independent task**	
Kaya et al. (2018)	8	19	SVM	5	Random split (63-27-10%)	43.00
Zahra et al. (2022)	8	19	CNN	5	10-fold	57.50
**This study**	8	19	SVM	6	5-fold	34.48
**Subject-dependent task**	
Kaya et al. (2018)	8	19	SVM	5	Random split (63-27-10%)	20.00–60.00
Anam et al. (2019)	4	19	RF	5	5-fold	51.00–56.00
Anam et al. (2020)	4	19	ADL	5	5-fold	74.61–77.75
Kato et al. (2020)	8	19	SVM	5	10-fold	23.90–58.30
Mwata-Velu et al. (2021)	8	4	BLS	5	200-fold	66.00–76.13
Azizah et al. (2022)	8	4	SVM	5	10-fold	21.20–66.60
Mwata-Velu et al. (2022)	4	4	EEGNet	5	200-fold	80.10–91.70
**This study**	8	19	EL	6	5-fold	35.83–55.00

*N* stands for the “number of subjects,” *n* stands for the “number of EEG channels,” *c* stands for the “number of classes,” and CV stands for the “Cross-Validation Method.” Classifiers are CNN, convolutional neural network; RF, random forest; ADL, autonomous deep learning; SVM, support vector machine; EEGNet, EEGNet deep learning model; BLS, bi-layered long-short classifier; EL, ensemble learning.

There are a few aspects that distinguish this study from previous studies in this field. These distinctional aspects to it, together with the contributions of this study to the literature can be explained as follows:

- ITD-based feature extraction study is conducted for FM classification. The first three higher frequency components and their different combinations were evaluated and their success rates were investigated with respect to different classifiers separately. In addition to the ITD-based features, EEG-based features have been evaluated without ITD decomposition to analyze the impact of the suggested ITD-based process. The observed results reveal that the highest performance values are mostly achieved in ITD-based approaches. Among ITD approaches, the most successful feature set is the first three PRC combinations (PRCs1-to-3).
- Additionally, the statistical significance-based feature selection process was applied to the first three PRC combinations. It has been observed that the performance of the classifier increases further in classifications performed using the first three PRC combinations. Thereby, in this study, the highest accuracy value was obtained by applying the combination of the first three modes to the Ensemble Learning classifier with ANOVA-based feature selection.
- To the best of our knowledge, our study presents the first approach where different combinations of PRCs were decomposed through ITD, and various features are utilized together to classify FM of EEG signals,
- We used both EEG signals of all subjects (eight subjects) and all channels (19 channels) of their EEG data in analyses, hence, excluding study design limitations (e.g., number of channels) to perform effective comparisons,
- Furthermore, this study is advantageous in all its stages (ITD-based feature extraction, and classification) in terms of workload and does not contain any complexity in the classification stage as in deep learning structures.
- Finally, we carried out a 6-class classification of FM by including the NoMT condition in FM in order to realize a more realistic BCI design and application for paralyzed patients. Such a design choice is crucial since it does not exclude occurrences of the Midas Touch Problem (Velichkovsky et al., 1997), which is actually the misinterpreted intention of interactive action fired by the interface. In the case of BCI development, when NoMT is discarded, it might easily cause Midas Touch occurrences to become the source of false positives and cause classification performance to degrade dramatically.

## 5 Conclusion

The accurate decoding of FM is accepted as a challenging task because the fingers are smaller than other limbs such as arms and hands and have a noisy signal nature. As a result, it is a more complicated task to discriminate among FM. In this study, an ITD-based machine learning approach is proposed for rapid and accurate classification of FM by using multi-channel EEG signals. Nineteen channel EEG data collected from eight subjects are used in our analysis. Firstly, the different modes are extracted from EEG signals using the ITD. The different features such as power, mean, sample entropy, high-frequency moments (first moment, second moment, third moment, fourth moment), and Hjorth parameters (activity, mobility, complexity) are evaluated using the first three modes of EEG signals. The single version of these modes and their different combinations are investigated in our suggested study, separately. Finally, FM classification through these extracted feature sets is performed using eight different machine-learning algorithms. Basically, we compared the performances of EEG-based features and the features extracted using the ITD algorithm. The experimental results reveal that the highest performance values are mostly (six out of eight classifier algorithms) acquired in ITD-based approaches. Additionally, the combinations of different modes mostly obtain the highest performance. Among all the different combinations, the first three combinations form the most successful feature set, and the highest accuracy values are achieved using this combination. On the other hand, the effectiveness of the ANOVA-based feature selection method is also investigated in this study. The results demonstrate that ANOVA-based feature selection improves the classifier performance by making it possible to find out the more discriminatory and relevant features. Among the classifier algorithms, the Ensemble Learning classifier appears to be the most successful classifier algorithm tested in this study. Therefore, in this study, the highest accuracy value of 55.00% is obtained in S4 (Subject E) by applying the combination of the first three modes to the Ensemble Learning classifier with ANOVA-based feature selection. The accuracy rates of subject-dependent analyses performed according to the Ensemble Learning classifier are found between 35.83 and 55.00% using the first three modes' combination (PRCs1-to-3) with ANOVA-based feature selection.

## Data availability statement

Publicly available datasets were analyzed in this study. This data can be found here: https://www.nature.com/articles/sdata2018211.

## Ethics statement

Ethical approval was not required for the study involving humans in accordance with the local legislation and institutional requirements. Written informed consent to participate in this study was not required from the participants or the participants' legal guardians/next of kin in accordance with the national legislation and the institutional requirements.

## Author contributions

MD: Formal analysis, Investigation, Methodology, Validation, Writing—original draft. YY: Formal analysis, Investigation, Supervision, Writing—original draft, Writing—review & editing. MP: Writing—original draft, Writing—review & editing, Funding acquisition. YI: Conceptualization, Methodology, Supervision, Writing—original draft, Writing—review & editing.
